# Arnicolide D Inhibits Triple Negative Breast Cancer Cell Proliferation by Suppression of Akt/mTOR and STAT3 Signaling Pathways

**DOI:** 10.7150/ijms.46925

**Published:** 2020-06-15

**Authors:** Zhao Qu, Yushan Lin, Daniel Kam-Wah Mok, Qingya Bian, William Chi-Shing Tai, Sibao Chen

**Affiliations:** 1 State Key Laboratory of Chinese Medicine and Molecular Pharmacology (Incubation), Shenzhen Research Institute, The Hong Kong Polytechnic University, Shenzhen 518057, China.; 2 Department of Applied Biology & Chemical Technology, The Hong Kong Polytechnic University, Hung Hom, Hong Kong S.A.R., China.; 3 Institute of Medicinal Plant Development, Chinese Academy of Medical Sciences & Peking Union Medical College, Beijing 100193, China.

**Keywords:** Arnicolide D, Triple negative breast cancer, Akt/mTOR, STAT3

## Abstract

Triple-Negative Breast Cancer (TNBC) is a most dangerous breast cancer subtype. The naturally occurring sesquiterpene lactone, arnicolide D (AD), has proven effective against a variety of tumors, however, the inhibitory effects of AD against TNBC and the underlying mechanisms remain unclear. In the present study, two TNBC cell lines (MDA-MB-231 and MDA-MB-468) and an MDA-MB-231 xenograft mouse model were employed to investigate the anti-TNBC effects of AD* in vitro* and* in vivo*. Cell viability was assessed by MTT assay. Cell cycle arrest and apoptosis were analyzed by flow cytometry. Protein levels were determined by immunoblotting. *In vitro* studies demonstrated that AD significantly decreased cell viability, and induced G2/M cell cycle arrest and apoptosis. *In vivo* assays showed that oral administration of 25 or 50 mg/kg AD for 22 days led to a reduction of tumor weights by 24.7% or 41.0%, without appreciable side effects. Mechanistically, AD inhibited the activation of Akt/mTOR and STAT3 signaling pathways. Based on our findings, AD is a promising candidate for development as an adjunctive therapeutic drug for TNBC.

## Introduction

Breast cancer is the most common malignancy in women worldwide. According to the World Health Organization International Agency for Research on Cancer, there were approximately 2.1 million newly diagnosed female breast cancer cases in 2018, accounting for around 25% cancer cases among women worldwide 1. Triple-Negative Breast Cancer (TNBC) is characterized by a lack of the estrogen receptor (ER), progesterone receptor (PR) and human epidermal growth receptor 2 (HER-2) that are commonly found in breast cancer, and accounts for approximately 15% - 20% of all breast cancer cases 2. TNBC tumors do not respond to endocrine or anti-HER2 therapies, and the systemic treatment is limited to cytotoxic chemotherapy 3. Furthermore, TNBC is associated with a high risk of relapse and metastasis, short progression-free survival, and a lower overall survival when compared to other breast cancer subtypes 4. Therefore, novel therapeutic drugs are necessary to combat TNBC.

**Figure 1:**
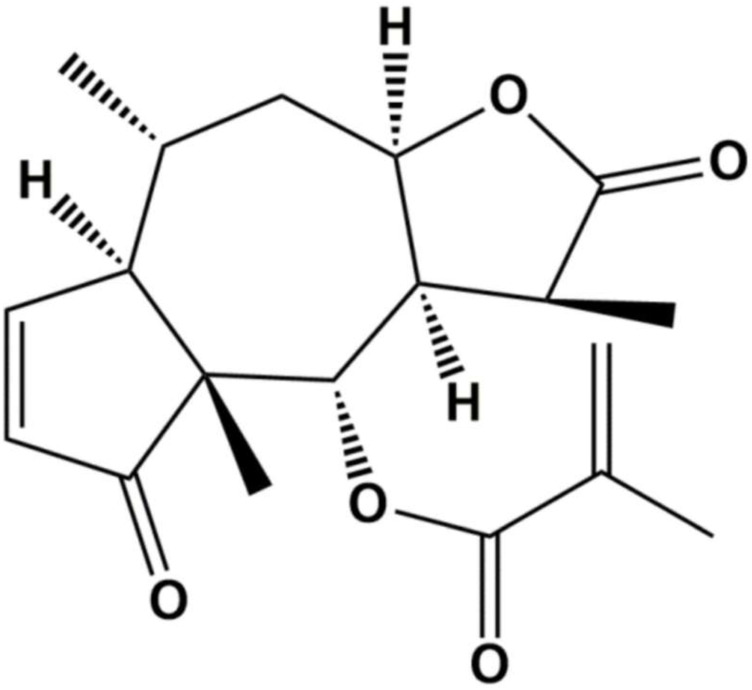
Chemical structure of Arnicolide D.

Natural products are an important source of drugs due to their abundance and high chemical structural diversity. Increasing amounts of research have shown that a variety of natural products can suppress proliferation of TNBC cells by blocking the cell cycle, inducing apoptosis, and inhibiting angiogenesis and migration 5-8. Thus, natural products are attracting increasing amounts of attention from researchers in the cancer field. Arnicolide D (AD) (Figure 1), a natural sesquiterpene lactone extracted from *Centipeda minima* 9, has been reported to exhibit anti-bacterial 9 and anti-cancer effects, in cancers including colon carcinoma 10, melanoma 11, and nasopharyngeal carcinoma 12. However, there have been no reports on the effects of AD in TNBC. In this study, we aimed to investigate the anti-cancer effects of AD in TNBC. In addition, the underlying molecular mechanisms were also explored.

## Materials and Methods

### Materials

Arnicolide D (C_19_H_24_O_5_, CAS34532-68-8) was purchased from Jiangsu Yongjian Pharmaceutical Co., Ltd. (Jiangsu, China). The purity of Arnicolide D was over 98% as analysed by high-performance liquid chromatography.

### Cell culture

TNBC cell lines MDA-MB-231 and MDA-MB-468 and the non-TNBC cell line MCF7 were purchased from the American Type Culture Collection (ATCC, Manassas, VA, USA), and maintained in DMEM containing 10% heat-inactivated FBS in a 37°C, 5% CO_2_ and humidified incubator. All cell lines were used within 20 passages and 2 months after resuscitation, and mycoplasma testing was routinely performed using the PCR Mycoplasma Detection Kit (TransGen Biotech, Beijing, China).

### Cell viability assay

To investigate the inhibitory effects of AD on breast cancer cell viability, 5×10^3^ (MDA-MB-231 and MCF-7) or 1×10^4^ (MDA-MB-468) cells were seeded into a 96-well plate and incubated for 24 h. Cells were then exposed to different concentrations of AD for 24, 48 or 72 h. Cells without treatment were used as a control. Cell viability was measured using MTT (Sigma-Aldrich), following manufacturer's protocols. IC_50_ values of AD were used to evaluate the *in vitro* anti-cancer cytotoxicity.

### Cell cycle and apoptosis analysis by flow cytometry

MDA-MB-231 and MDA-MB-468 cells were seeded in 6-well plates and treated with AD for 24 or 48 h. Cell pellets were collected and centrifuged at 1000 rpm for 5 min. For the cell cycle analysis, cells were fixed with ice-cold 70% ethanol at 4°C overnight, then stained with PI (Beyotime, Shanghai, China) and measured using a BD Accuri C6 flow cytometry system (Becton Dickson Immunocytometry-Systems, San Jose, CA, USA) and the data analyzed by ModFit software (ModFit LT 5.0, Verity Software House, Inc., Topsham, ME, USA). For apoptosis analysis, cell pellets were stained with FITC-labeled Annexin V and PI and assessed immediately using a CytoFLEX flow cytometer (Beckman Coulter, Brea, CA, USA) analysis immediately.

### Hoechst 33342 staining

Briefly, the cells were treated with AD for 24 h. Following washes with PBS, cells were fixed with 4% paraformaldehyde, stained with Hoechst 33342 for 15 min at room temperature, and then examined by fluorescence microscopy (Nikon, Tokyo, Japan).

### Wound-healing assay

Cell migration was evaluated by wound-healing assay. MDA-MB-231 cells (4×10^4^ cells/well) were seeded into 35 mm high μ-dishes with culture inserts (ibidi, Germany). Following removal of culture inserts and washes with medium, cells were treated with various concentrations of AD, or batimastat (Aladdin, Shanghai, China) as a positive control. Migration of cells into the wound area was photographed under a microscope at 0, 6, 12, 24 and 48 h time points. The scratch open area was calculated using ImageJ software.

### Western blot assay

Total protein was extracted using cell lysis RIPA buffer (Cell signaling technology, Beverly, MA, USA) and protein concentrations were determined by BCA Protein Assay (Thermo Scientific). Protein samples (10 μg per well) were subjected to 10% - 15% SDS-PAGE and then wet-transferred to PVDF membranes. The blots were then blocked in 5% non-fat milk and incubated with primary antibodies in 5% BSA overnight. HRP-conjugated secondary antibodies were used following washes with TBST buffer. Protein bands were detected using ECL western blotting substrate (Tanon, Shanghai, China). QuantityOne software (Bio-Rad, Hercules, California, USA) was used for band density analysis.

### Tumor xenograft study

6 week-old female BALB/c nude mice were obtained from Beijing Vital River Laboratory Animal Technology Co., Ltd. (Beijing, China). All animal experimental procedures were performed according to the Institutional Guidelines and Animal Ordinance of the Department of Health, and approved by the Hong Kong Polytechnic University Animal Subjects Ethics Sub-committee.

Mice were inoculated with MDA-MB-231 cells (5×10^6^) in the fourth mammary fat pad. After two weeks inoculation, the average tumor volume reach to 70 mm^3^, mice were randomly divided into four groups (6 mice per group), and treated by oral gavage with 25 or 50 mg/kg/day of AD, or an equivalent amount of solvent (0.5% CMCNa, 1% Tween-80, 10 mL/kg) as a negative control (vehicle group). A positive control group received 10 mg/kg of docetaxel once a week by intraperitoneal (i.p.) injection. Body weight and tumor size were measured every two or three days during the experimental period. Tumor size was monitored using calipers and tumor volumes were calculated using the formula volume = (length × width^2^)/2. Treatment duration was 22 days, and the average tumor volume of the vehicle group reached 800 mm^3^ during that time. At the experimental endpoint, all mice were sacrificed and their tumors and vital organs were harvested and weighed.

Formalin fixed tumor tissues were embedded with paraffin, and 5 μm sections were cut serially and stained with hematoxylin and eosin (H&E). Immunohistochemical detection of Ki67 was performed using the SPlink Detection Kit (ZSGB-BIO, Beijing, China), following manufacturer's protocols. In briefly, sections were deparaffinized and rehydrated, antigen were retrieved by heating in citric acid solution. Then, sections were incubated with H_2_O_2_ to quench endogenous peroxidase activity, blocked with normal goat serum working solution, incubated with primary antibody against Ki67 (1:1000 dilution; Proteintech, Wuhan, Hubei, China) at 4°C overnight. After rinsing three times with PBS, sections were incubated with biotin-labeled goat anti-rabbit IgG and horseradish peroxidase-labeled streptavidin. A final incubation in DAB chromogenic reagent was performed for visualization. All sections were stained with hematoxylin, dehydrated, cleared, coverslipped and photographed.

### Statistical analysis

All data are presented as means ± SEM and analysed using one-way analysis of variance (ANOVA) followed by LSD post hoc or Dunnett's tests. Differences were considered to be statistically significant at P < 0.05.

## Results

### AD inhibited the cell proliferation of breast cancer cells

**Figure 2:**
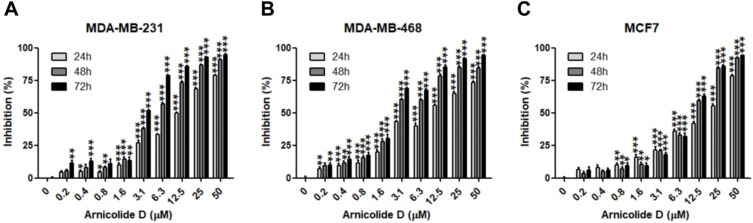
AD inhibited breast cancer cell proliferation. Cell viability of (A) MDA-MB-231, (B) MDA-MB-468, and (C) MCF7 was measured by MTT assay after AD treatment at the indicated concentrations for 24, 48 and 72 h. Data are presented as means ± SEM from three independent experiments. *P<0.05, **P<0.01, ***P<0.001, compared to control.

The anti-proliferative effects of AD in breast cancer cells were examined by MTT assay in two TNBC cell lines, MDA-MB-231 and MDA-MB-468, as well as the ER and PR positive cell line MCF7. Results showed that AD exhibited anti-cancer activity in a dose- and time-dependent manner (Table 1, Figure 2). IC_50_ values of AD in MDA-MB-231, MDA-MB-468, and MCF7 were 12.04, 9.507, and 15.19 μM for 24 h treatment, 5.211, 3.405, and 9.083 μM for 48 h treatment, and 3.258, 2.515, and 8.909 μM for 72 h treatment, respectively. The inhibitory effects of AD in the TNBC cell lines MDA-MB-231 and MDA-MB-468 were superior to the non-TNBC cell line MCF7 after 48 and 72 h treatments.

### AD induced cell cycle arrest in TNBC cells

**Figure 3:**
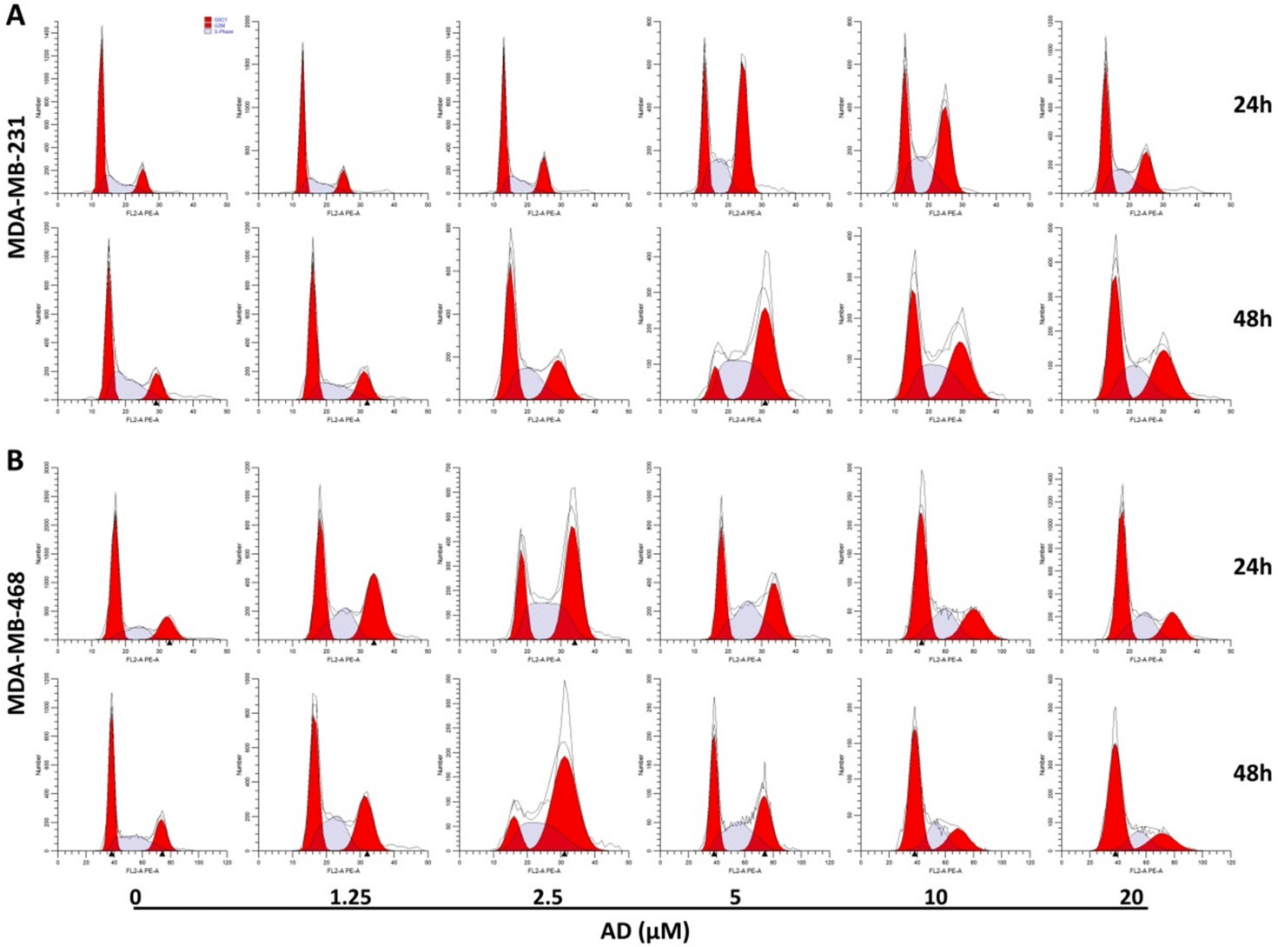
AD induced cell cycle arrest in TNBC. (A) MDA-MB-231 and (B) MDA-MB-468 cells were stained with PI and the cell cycle analyzed by flow cytometry.

To elucidate the underlying mechanisms of AD-induced inhibition, cell-cycle distribution patterns were analysed using flow cytometry in MDA-MB-231 and MDA-MB-468. As shown in Figure 3A, the proportion of MDA-MB-231 cells in the G2/M phase was significantly increased after 5 μM AD treatment for both 24 and 48 h, whereas higher doses of AD exhibited weaker inhibition of the cell cycle. Similarly, AD significantly increased MDA-MB-468 cell cycle arrest at the G2/M phase at a concentration of 2.5 μM (Figure 3B). These results suggested that the anti-proliferative effect of AD may be through cell cycle arrest, combined with other mechanisms at higher doses.

### AD induced apoptosis in TNBC cells

**Figure 4:**
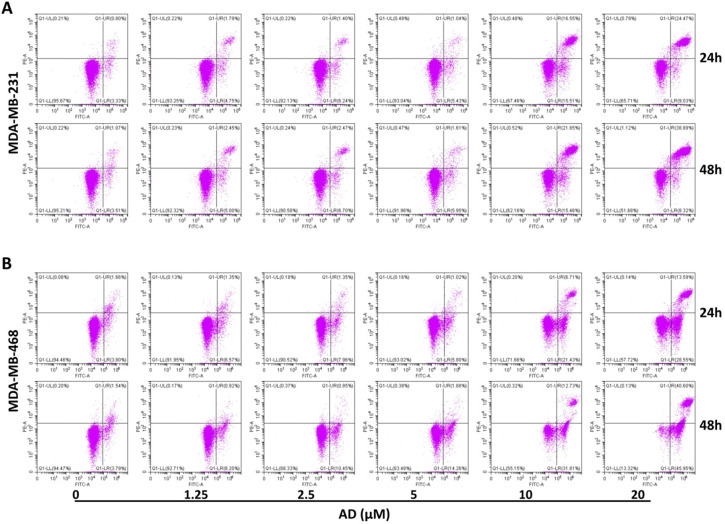
AD induced apoptosis in TNBC. (A) MDA-MB-231 and (B) MDA-MB-468 were treated with AD, stained with AnnexinV-FITC/PI, and cell apoptosis analyzed by flow cytometry.

Apoptosis is another important factor contributing to cell proliferation. Thus, we measured apoptosis in TNBC cells with flow cytometry after AnnexinV-FITC/PI staining. Results showed that AD significantly increased the proportion of apoptotic cells at doses of 10 and 20 μM (Figure 4). In MDA-MB-231, the percentage of Annexin V positive cells increased by 27.93%, and 29.37% after 24 h treatment, and 32.43%, and 42.63% after 48 h treatment, respectively. In MDA-MB-468, the percentage of apoptotic cells increased by 22.68%, and 36.68% after 24 h treatment, and 39.21%, and 81.22% after 48 h treatment, respectively. These results indicated that low doses of AD led to cell cycle arrest, and as concentrations increased, eventually apoptosis.

**Figure 5:**
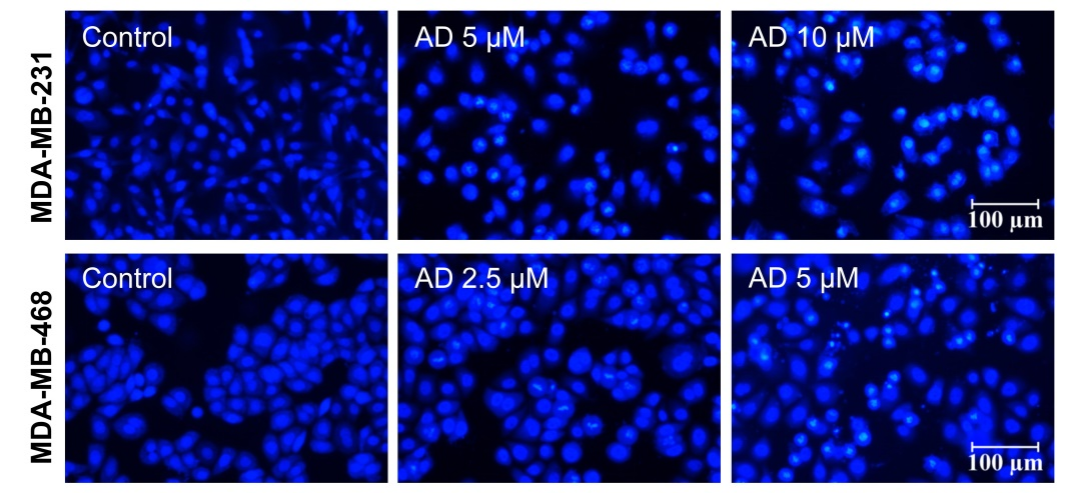
AD induced apoptotic body formation. MDA-MB-231 and MDA-MB-468 cells were treated with AD for 24 h, stained with Hoechst 33342, and nuclear morphology was photographed using an inverted fluorescence microscope (200x). Scale bar 100 µm.

To observe changes in the nuclear morphology of MDA-MB-231 and MDA-MB-468 cells, cells were photographed under an inverted fluorescence microscope after staining with Hoechst 33342. As shown in Figure 5, distinct chromatin condensation and formation of apoptotic bodies was observed after 24 h treatment of AD.

### AD inhibited cell migration in MDA-MB-231 cells

**Figure 6:**
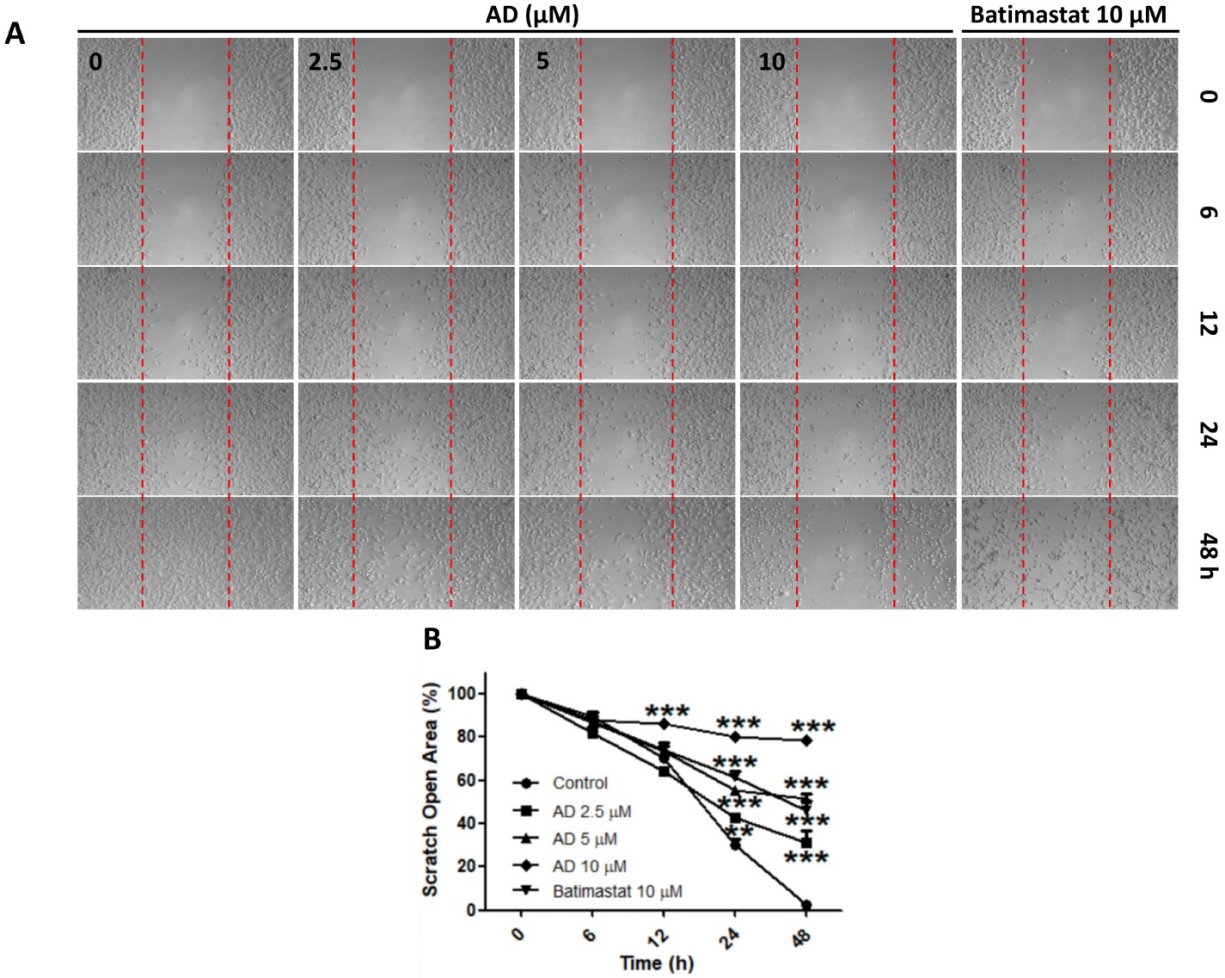
AD inhibited cell migration in MDA-MB-231 cells. (A) Cell migration was measured by wound-healing assay. (B) Quantification of scratch open area. Data are present as means ± SEM from three independent experiments. *P<0.05, **P<0.01, ***P<0.001, compared to control.

Due to the highly invasive characteristic of TNBC, using the wound-healing assay, we investigated the inhibitive efficacy of AD on invasion in MDA-MB-231 cells. Results demonstrated that AD significantly suppressed cell migration at doses of 2.5, 5, and 10 μM after 24 and 48 h of treatment (Figure 6).

### AD inhibited Akt/mTOR signaling in TNBC cells

**Figure 7:**
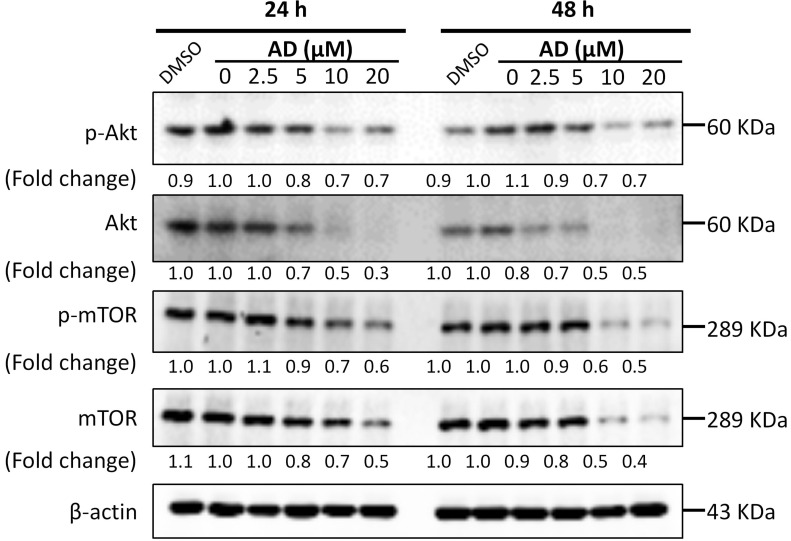
AD inhibited Akt/mTOR signaling in TNBC cells. MDA-MB-231 cells were treated with AD for 24 or 48 h, collected and immunoblotted with the indicated antibodies. β-actin was used as an internal control.

Activation of the PI3K/Akt/mTOR signaling pathway plays a significant role in controlling cell growth, proliferation and metastasis, and has been identified as an important potential therapeutic target in breast cancer 13. Therefore, we examined the key proteins of this pathway in MDA-MB-231 cells. As shown in Figure 7, AD significantly inhibited the expression of Akt and mTOR, as well as their phosphorylated forms. These data indicated that AD has a direct inhibitory effect on the PI3K/Akt/mTOR pathway.

### AD inhibited STAT3 signaling in TNBC cells

**Figure 8:**
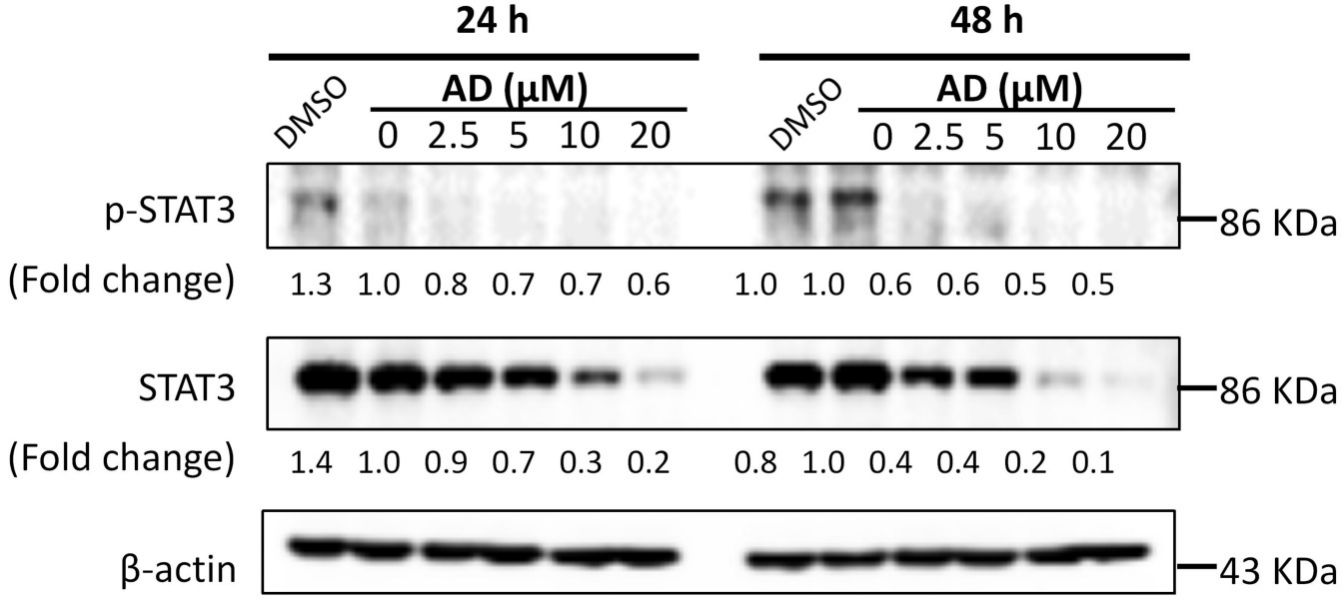
AD inhibited STAT3 signaling in TNBC cells. MDA-MB-231 cells were treated with AD for 24 or 48 h, collected and immunoblotted with the indicated antibodies. β-actin was used as an internal control.

STAT3 is an important transcription factor. Its overexpression and constitutive activation play a key role in the progression, proliferation and metastasis of breast cancer 14. Therefore, expression levels of STAT3 and p-STAT3 were examined by Western-blotting after AD treatment. As shown in Figure 8, AD significantly downregulated the expression of STAT3 and p-STAT3 in a dose-dependent manner in MDA-MB-231 cells.

### AD inhibited *in vivo* MDA-MB-231 xenograft tumor growth

**Figure 9:**
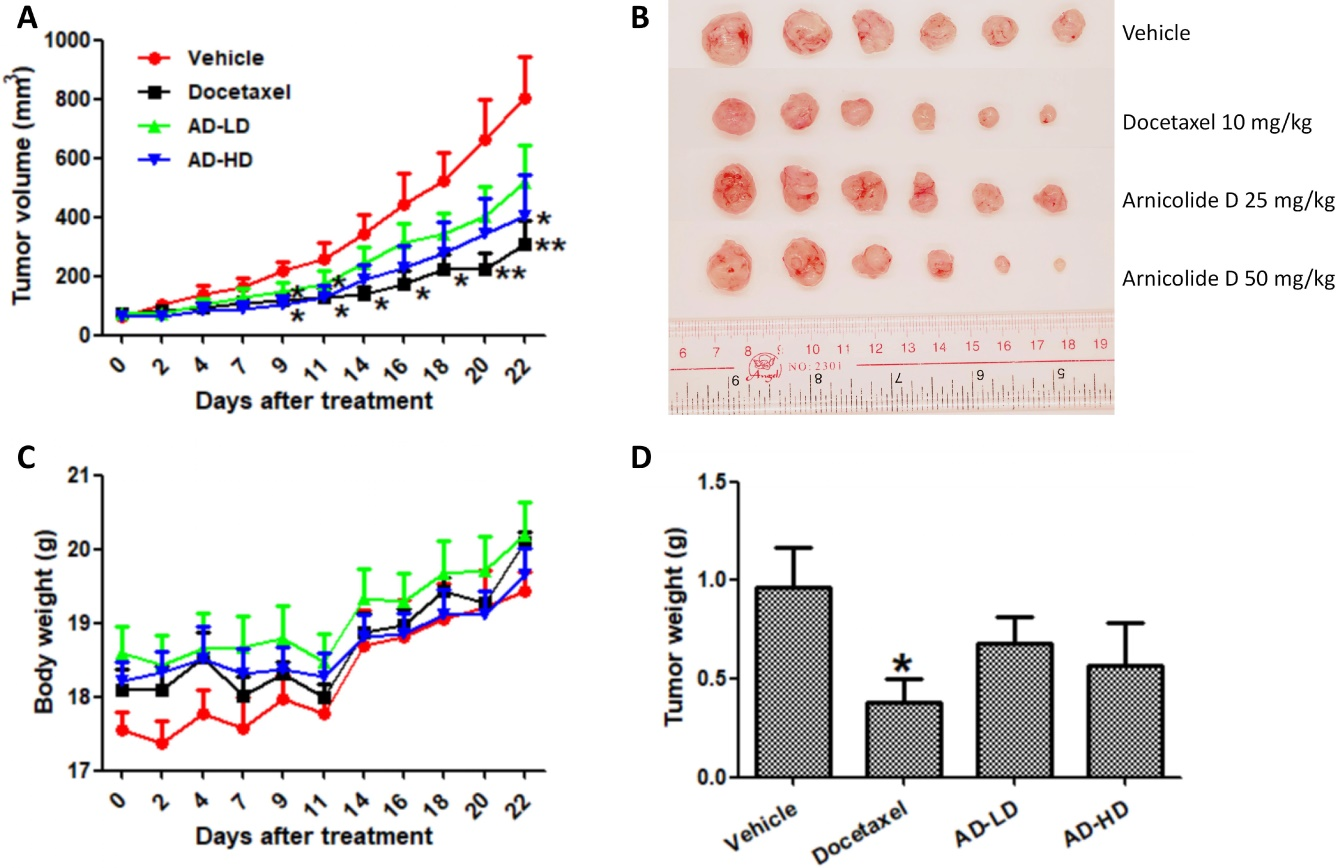
AD inhibited *in vivo* MDA-MB-231 xenograft tumor growth. Female BALB/c nude mice were inoculated with MDA-MB-231 cells in their mammary fat pads. Two weeks later, mice were randomized into four groups and received treatment with vehicle (0.5% CMCNa, 1% Tween-80), AD-LD (AD 25 mg/kg), or AD-HD (AD 50 mg/kg) daily via oral adminstration, or docetaxel (10 mg/kg) once a week via i.p. injection. The treatment period lasted for 22 days after which all mice were sacrificed. (A) Tumor volumes of each group throughout the experimental period. (B) Images of tumors at experimental endpoint. (C) Mouse body weights throughout the experimental period. (D) Tumor weights at experimental endpoint. Data are expressed as means ± SEM.*P<0.05, **P<0.01, compared to the vehicle group.

Based on the above results, we employed an orthotopic xenograft mouse model to investigate the *in vivo* anti-cancer effect of AD. Mice received oral treatment with AD at a low (25 mg/kg) or high (50 mg/kg) doses, or i.p. injection of docetaxel as a positive control. At day 22, average tumor volumes were reduced by 35.1% (AD 25 mg/kg), 49.5% (AD 50 mg/kg), and 61.7% (docetaxel) when compared to vehicle control group (Figure 9A-B). Average tumor weights were reduced by 24.7% (AD 25 mg/kg), 41.0% (AD 50 mg/kg) and 60.4% (docetaxel) when compared to control (Figure 9D). During the experimental period, AD did not cause significant body weight loss (Figure 9C).

**Figure 10:**
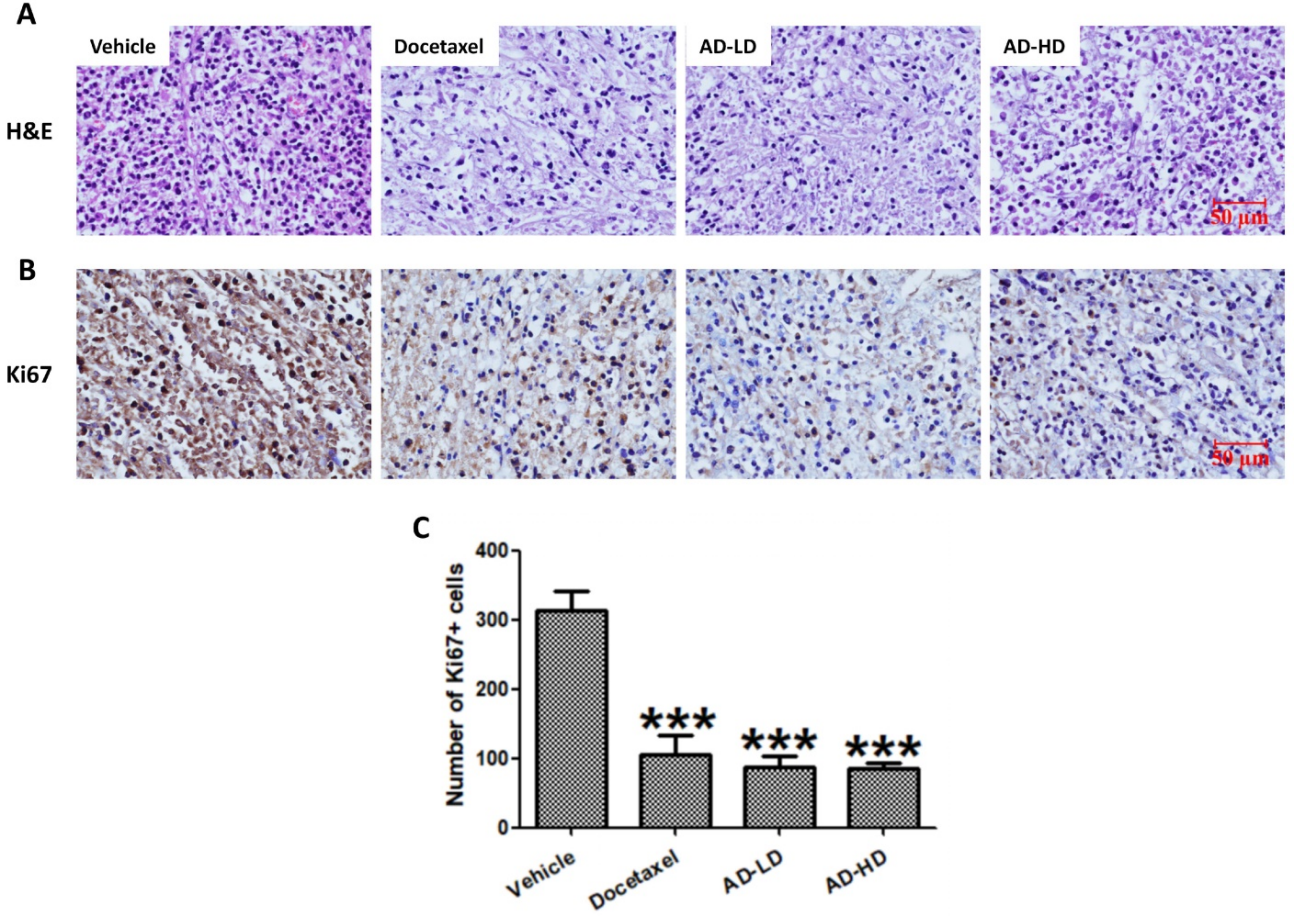
AD inhibited *in vivo* cancer cell proliferation. Tumors were stained with (A) H&E and (B) Ki67. Representative images are shown (200x). Scale bar, 50 µm. (C) Number of Ki67 positive cells are expressed as Means ± SEM. ***P<0.001, compared to the vehicle group.

To assess the changes in tumor morphology, the tumor sections were stained with H&E. Similar to the docetaxel-treated group, the density of tumor cells in AD-treated group were notably decreased when compared to the vehicle group (Figure 10). We then further, examined the expression of cell proliferation marker Ki67. As shown in Figure 10, AD inhibited the expression of Ki67 when compared to the vehicle group. The above results indicated that AD exhibited *in vivo* anti-TNBC effects.

## Discussion

TNBC exhibits a more aggressive phenotype and poorer prognosis than other breast cancer subtypes 4. Natural products are a rich source for drug discovery of compounds to combat TNBC. In this study, we demonstrated that AD suppressed TNBC growth, *in vitro* and *in vivo*. AD exhibited inhibitory effects on the TNBC cell lines MDA-MB-231 and MDA-MB-468, and with a higher efficacy than in the non-TNBC cell line MCF7. Furthermore, oral administration of AD effectively inhibited xenograft tumor growth *in vivo* without apparent side effects.

Mitotic catastrophe and apoptosis are considered as two major types of cell death in cancer therapy 15. In this study, we found that AD exhibited different effects at various concentrations. AD induced G2/M cell cycle arrest in MDA-MB-231 and MDA-MB-468 cells, while at higher doses, AD also exhibited significant apoptosis-inducing effects.

The PI3K/AKT/mTOR pathway regulates cell proliferation and survival, and is over-activated in more than 60% of TNBC patients 16. In addition, p-mTOR expression is associated with poor prognosis in early-stage TNBC 17. In recent years, the PI3K/Akt/mTOR pathway has been considered as a therapeutic target for novel drug discovery and several pathway inhibitors such as PQR309, Ipatasertib, AZD5363, and temsirolimus are being evaluated in clinical trials 18. In this study, we found that AD downregulated the expression of AKT and mTOR in TNBC cells. Our results suggested that the inhibition of PI3K/AKT/mTOR pathway may be a potential mechanism for the anti-TNBC activity of AD.

STAT3 is constitutively activated in more than 40% of breast cancers and is associated with initiation, progression, metastasis, chemoresistance, and immune evasion of TNBC 19, 20. Several natural sesquiterpene lactones, such as alantolactone, parthenolide and dehydrocostuslactone, exhibit anti-TNBC effects via downregulation of p-STAT3 expression 21. In the present study, we found that AD inhibited the expression of STAT3 in MDA-MB-231 cells, which implies that STAT3 may be involved in the AD-mediated inhibition of TNBC cells as well.

A prior clinical study has reported that the mTOR and JAK2/STAT3 pathways are over-activated in inflammatory and invasive ductal breast cancers after neo-adjuvant chemotherapy 22. Hence, the combination of therapy against mTOR and STAT3 is a promising prospect for the treatment of triple negative breast cancer 23. In this study, AD exhibited significant inhibition of the AKT/mTOR and STAT3 pathways, and may be a promising drug candidate against TNBC.

In conclusion, this study demonstrates for the first time that AD could inhibit TNBC cell growth both *in vitro* and *in vivo*, and the mechanism of these effects was associated or at least partially associated with the inhibition of the Akt/mTOR and STAT3 signaling pathways. Our findings suggest AD as a potential novel adjunctive therapeutic drug for TNBC.

## Figures and Tables

**Table 1:** IC_50_ values of AD in human breast cancer cell lines (µM)

Cell line	24 h	48 h	72 h
MDA-MB-231	12.04	5.211	3.258
MDA-MB-468	9.507	3.405	2.515
MCF7	15.19	9.083	8.909
